# Adaptivity: a path towards general swarm intelligence?

**DOI:** 10.3389/frobt.2023.1163185

**Published:** 2023-05-09

**Authors:** Hian Lee Kwa, Jabez Leong Kit, Nikolaj Horsevad, Julien Philippot, Mohammad Savari, Roland Bouffanais

**Affiliations:** ^1^ Thales Research and Technology, Singapore, Singapore; ^2^ Engineering Product Design, Singapore University of Technology and Design, Singapore, Singapore; ^3^ Mechanical Engineering, University of Ottawa, Ottawa, ON, Canada

**Keywords:** adaptivity, collective robotics, multi-agent systems, multi-robot systems, swarm robotics, swarm intelligence

## Abstract

The field of multi-robot systems (MRS) has recently been gaining increasing popularity among various research groups, practitioners, and a wide range of industries. Compared to single-robot systems, multi-robot systems are able to perform tasks more efficiently or accomplish objectives that are simply not feasible with a single unit. This makes such multi-robot systems ideal candidates for carrying out distributed tasks in large environments—e.g., performing object retrieval, mapping, or surveillance. However, the traditional approach to multi-robot systems using global planning and centralized operation is, in general, ill-suited for fulfilling tasks in unstructured and dynamic environments. Swarming multi-robot systems have been proposed to deal with such steep challenges, primarily owing to its adaptivity. These qualities are expressed by the system’s ability to learn or change its behavior in response to new and/or evolving operating conditions. Given its importance, in this perspective, we focus on the critical importance of adaptivity for effective multi-robot system swarming and use it as the basis for defining, and potentially quantifying, swarm intelligence. In addition, we highlight the importance of establishing a suite of benchmark tests to measure a swarm’s level of adaptivity. We believe that a focus on achieving increased levels of swarm intelligence through the focus on adaptivity will further be able to elevate the field of swarm robotics.

## 1 Introduction

The field of multi-robot systems (MRS) has recently been gaining popularity among various research groups, practitioners, and industrial actors, seeking productivity gains through advanced automation. MRS are able to perform tasks more efficiently and effectively and accomplish missions that, in some cases, are simply out of reach for a single unit. This makes MRS the ideal candidate for carrying out distributed tasks in large and rapidly changing environments—e.g., performing object retrieval, search-and-rescue, and surveillance (Darmanin and Bugeja, 2017). However, the classical centralized approach to MRS can rapidly become unfeasible when dealing with tasks in unstructured and/or dynamic environments. Swarming MRS have been utilized to address some of these shortcomings and challenges, given that swarming robots exhibit the highly desirable features of flexibility, robustness, and scalability, thereby giving a system’s agents to carry out complex tasks as a collective unit (Dorigo et al., 2021). Moreover, given the ease of mass production of robotic units, many researchers and industries alike are turning toward the use of low-cost and easy-to-manufacture robotic units in swarms (Bouffanais, 2016). The versatility of robot swarms is attested by a wealth of applications spanning from environmental monitoring (Thenius et al., 2016; Vallegra et al., 2018; Zoss et al., 2018) to area mapping (Kit et al., 2019; Mitchell and Michael, 2019) and area defense (Strickland et al., 2018; Shishika and Paley, 2019).

The popularity of swarming MRS can partly be credited to the three main advantages that such systems have over their centralized MRS counterparts: scalability, robustness, and flexibility. Of the three key properties, the word “flexibility” has often been used interchangeably with “adaptivity.” However, as defined by Dorigo et al. (2021), flexibility is the system’s capacity to perform tasks that depart from those chosen at design time, while adaptivity is the system’s capacity to learn or change its behavior to respond to new operating conditions. In its current state, swarming MRS have been demonstrated to be flexible; swarms using the same strategies and behavioral parameters have demonstrated their ability to carry out a given task over a wide range of scenarios (Esterle, 2018a; Kit et al., 2019), albeit with varying levels of performance.

Despite their flexibility, current swarms have only shown limited levels of adaptivity; the development of swarm strategies and behaviors has mostly been limited to optimizing a system to fit a narrow range of pre-specified environmental conditions. This has been achieved through the modification of various behavioral and strategy parameters, according to locally or globally measured metrics, and endows the swarming MRS with what could be qualified as “narrow” adaptivity (Pang et al., 2019; Birattari et al., 2020; Nauta et al., 2020). Although doing so allows a swarm to adapt its behavior and maximizes its performance within a narrow range of conditions, the MRS will be unable to adequately operate should the environment vary outside these conditions or in an unexpected manner. A truly adaptive swarm, i.e., one that displays a “general” swarm intelligence, would be able to cope and adapt its behaviors to any conditions presented to it, and be able to achieve different types of tasks depending on an agent’s physical capabilities (e.g., one cannot expect an area mapping swarm to carry out an object retrieval task).

In this perspective, we give a preliminary definition of the concept of general swarm intelligence and address what we believe is required to achieve true adaptivity, i.e., a system’s ability to learn or change its behavior in response to new operating conditions. We also discuss the inherent challenges in evaluating a concept, such as adaptivity, and propose the use of a benchmark test suite for its quantification and comparison. Finally, we discuss what is needed for a general swarm intelligence algorithm to be obtained. In light of this, we firmly believe that future research in the field of swarm robotics should focus on making robotic swarms more adaptive, thereby increasing their viability.

## 2 General intelligence

### 2.1 Strong and weak artificial (swarm) intelligence

The key feature of flexibility that defines the effectiveness of MRS can be seen as reminiscent of “weak” or “narrow” AI. Consider the flexibility exhibited by a school of fish when it carries out a rapid evasive maneuver, following a predator’s attack: swarm intelligence at its best. Searle (1980) stated that with weak AI, “the principal value of the computer in the study of the mind is that it gives us a very powerful tool.” For our school of fish, the group’s collective escape strategy from its predators is indeed a powerful survival tool. However, this particular strategy would have to be adapted if it were to remain as effective and relevant for a different species; a flock of birds would have to use a different strategy, given that they live in different mediums and deal with different predators. In contrast, with artificial general intelligence (AGI)—also referred to as “strong” AI—“the computer is not merely a tool in the study of the mind: rather, the appropriately programmed computer really is a mind, in the sense that computers, given the right programs, can be literally said to understand and have other cognitive states” (Searle, 1980). As such, systems with AGI should be able to generalize their knowledge and use them in various different contexts (Goertzel, 2014). General swarm intelligence would then be akin to AGI, one in which a system demonstrates high levels of adaptivity, thereby allowing it to effectively function in any highly dynamic environment under any given set of circumstances.

It is worth reminding that AI is usually defined and constructed using some characteristics associated with human intelligence. Interestingly, natural swarm intelligence (SI) exhibited by animal groups constitutes yet another form of intelligence—albeit a collective and decentralized one—that can be seen as distinct from individual human intelligence. To better appreciate the concept of SI, it can be useful to consider each bird in a flock of birds as a neuron within a brain, with the intelligence emerging from the repeated interactions between the constituting units. As a matter of fact, the current framework for AI does not always lend itself to SI with its decentralized information gathering, social information transfer, and distributed processing. Nonetheless, there is broad consensus within the scientific community about SI being a particular subset of AI, as currently defined, without necessarily knowing where SI exactly fits (Bonabeau et al., 1999; Sadiku et al., 2021). It is important to note that the central difference between AI and SI is more than a simple question of application. The key distinction is that collective intelligence emerges from the actions of the swarm’s agents that ultimately lead to complex collective behaviors that are greater than the sum of its parts. The macro-level behavior cannot be directly programmed. Instead, the algorithm must be designed to empower individual agents to come together as an emergent swarm to accomplish a desired task. This extra layer of separation between the programming and the desired output behavior generally makes achieving swarm intelligence much more complicated than the equivalent AI behavior (Birattari et al., 2020; Nguyen et al., 2020; Bianconi et al., 2023).

Following this line of thought, the development of current non-adaptive swarming algorithms can be compared to that of current non-adaptive narrow AI algorithms. In both cases, some form of reprogramming or intervention is required by a human operator to match a system to its environment and/or task (Goertzel, 2014). This parallel leads us to believe that breakthroughs in AGI development will concomitantly lead to more adaptive outcomes for swarms. For instance, Butz (2021) argues that current (narrow) machine learning “optimizes the best possible strategy within the status quo” and that current algorithms are “reflective rather than prospective.” On the other hand, strong AI needs to be predictive to infer the hidden causes behind its observations and explain the reasoning behind its decision-making process. This offers us prospective routes to improve the current SI status quo.

### 2.2 Defining swarm intelligence

Currently, there is no concise and universally accepted definition of SI. Given the search for a definition of intelligence by both the SI and AI communities, we have taken inspiration from the various definitions proposed by the AI community to develop one for SI. To this end, we have made a selection of definitions from Legg and Hutter (2007) shown in Table 1. In this table, it can be seen that the key common element shared across definitions is a system’s ability to make necessary behavioral adjustments to cope with the changing environment and to achieve its goals that may vary over time. Together with the importance of collective behavior and adaptivity discussed previously, we believe that swarm intelligence can be defined as follows: “Swarm Intelligence is the emergent ability of a decentralized system of agents to make the appropriate adjustments to its collective behavior, thereby allowing the system to achieve changing goals in dynamic environments.”

**TABLE 1:** List of AI definitions selected from Legg and Hutter (2007).

Definition	Source
“Any system …that generates adaptive behavior to meet goals in a range of environments can be said to be intelligent.”	Fogel (1995)
“Achieving complex goals in complex environments.”	Goertzel (2006)
“Intelligence measures an agent’s ability to achieve goals in a wide range of environments.”	Legg and Hutter (2006)
“…in any real situation, behavior appropriate to the ends of the system and adaptive to the demands of the environment can occur, within some limits of speed and complexity.”	Newel and Simon (1976)
“(An intelligent agent does what) is appropriate for its circumstances and its goal, it is flexible to changing environments and changing goals, it learns from experience, and it makes appropriate choices, given perceptual limitations and finite computation.”	Poole et al. (1998)
“Intelligence is the ability of an information processing system to adapt to its environment with insufficient knowledge and resources.”	Wang (1995)

## 3 Adaptivity

### 3.1 Current state of the art

Our proposed definition of SI is strongly linked with a system’s level of adaptivity—i.e., the system’s ability to learn or change its behavior in response to new operating conditions (Dorigo et al., 2021). Despite the importance of adaptivity for swarm intelligence, the vast majority of research carried out on swarm robotic systems aims to optimize a strategy or a certain set of behavioral parameters to maximize its performance for a task-specific and/or environmental conditions. In these works, adaptivity is usually demonstrated as an afterthought, with systems exhibiting that they are able to function in various operating conditions, albeit with a certain degree of performance degradation (Coquet et al., 2019; Kwa et al., 2022a).

**FIGURE 1:**
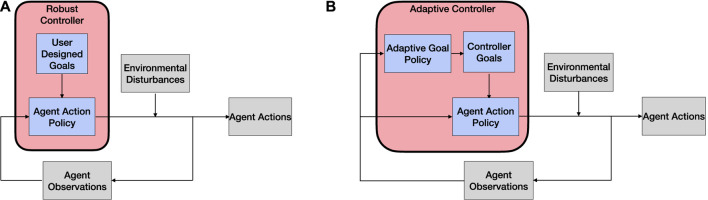
Flowchart of controllers used in swarming MRS to promote adaptivity. **(A)** Robust controllers. **(B)** Adaptive controllers.

Adaptive swarms can be achieved through either offline or online swarm design methods, as suggested by Birattari et al. (2020). These two radically different approaches toward adaptivity have also been described by Hasbach and Bennewitz (2022) as two different controllers—(1) a robust controller that allows a system to control its behavior when the environmental conditions vary within the expected range (i.e., offline methods) and (2) an adaptive controller that allows the system to implement rules and new system goals to adjust the robust controller when the fit between the robust controller and the situation set is not optimal (i.e., online design methods). A visual representation of the differences between these two controllers is shown in Figure 1.

Currently, adaptivity in swarms is mostly achieved using robust controllers. In doing so, swarms develop a base set of strategies that can be further optimized to maximize the system’s performance over a specific range of task conditions, thereby achieving narrow adaptivity. Such a system was used by Rausch et al. (2019), who identified an optimal degree of connectivity between agents and varied the communication ranges of the individual agents to maintain a consistent number of neighbors, thereby maintaining the coherence of the system. Similarly, by optimizing an individual agent’s movements to maintain the inter-agent spacing and a constant Voronoi cell area, Vallegra et al. (2018) and Kouzeghar et al. (2023) were able to maintain a high level of coverage of two different types of dynamic environments using swarms composed of autonomous buoys and UAVs, respectively. Using this approach, systems will be able to perform well within a specific range of conditions. However, should the operating conditions vary outside this range—i.e., when a high level of adaptivity is demanded from the system—the performance of the swarm will inevitably suffer due to the fact that the system has been optimized prior to its implementation (Hunt, 2020).

### 3.2 Toward general adaptivity

We believe that attaining general swarm intelligence would require a system to have general adaptivity. This would, therefore, involve using adaptive controllers. In doing so, systems will be able to optimize, learn, or develop new behavioral and strategy parameters as the mission progresses, thereby allowing systems to display the sought-after general adaptivity. This naturally allows for a higher degree of adaptivity due to the increased abilities of an MRS to modulate and vary its behavior, better allowing the system to maintain its performance should the operating conditions change. It is worth noting that a few initial attempts have been made in using these methods, for instance, in systems making use of Lévy walks, individual agents autonomously varying their individual Lévy parameters to modulate the level of exploration and exploitation being carried out (Pang et al., 2019; Nauta et al., 2020; Garcia-Saura et al., 2021; Kwa et al., 2022a). Along the same vein, Esterle (2018b) developed a swarm robotic system with agents autonomously switching between two states, thereby allowing the system to track targets that continuously appear and disappear in the environment. This was achieved using a variable threshold value that was calculated based on the number of targets currently tracked and the number of targets to be tracked.

In making swarms more adaptive, regardless of the approach, system designers need to identify the correct set of cues to determine if adaptation should be triggered and how these cues will be sensed (Hunt, 2020). In addition, designers should also be wary of agents responding to false alarms when detecting these cues, i.e., systems adapting to changes in the operating conditions that have not actually taken place. This usually entails a process, where a system’s responsiveness to environmental changes needs to be balanced against its resilience to false alerts, also known as the stability–flexibility dilemma in neural systems (Liljenström, 2003; Wahby et al., 2019; Leonard and Levin, 2022).

## 4 Swarm intelligence benchmark framework

### 4.1 The need for a swarm intelligence benchmark

Given the need to push toward increasing levels of swarm intelligence, i.e., system adaptivity, we believe that a common benchmark is required to facilitate the comparison between different strategies, a belief also shared by Dorigo et al. (2021). The concept of benchmarking is rather common and already exists in other more mature research fields such as optimization, where algorithms are tested against various benchmark functions (Huband et al., 2006; Li et al., 2013). Similarly, various test environments have been created to evaluate control strategies developed by means of reinforcement learning algorithms for single agents (Duan et al., 2016; Cobbe et al., 2020) and multiple agents (Lowe et al., 2017). The introduction of such a benchmark for a system’s adaptivity has the potential to unify the research community by providing a basis for measurement and comparison, and allow practitioners to understand how high levels of adaptivity are derived.

Although swarm intelligent solutions have been established for multiple problems (Kit et al., 2019; Kwa et al., 2022b; Esterle and King, 2022; Kouzeghar et al., 2023), they are often evaluated based on their own operational performance for a specified scenario and fixed conditions. This is to be expected as such systems are developed and applied to solve a particular problem or carry out a specific task. Therefore, the evaluation of a system’s operational performances allows it to be compared to previous solutions and strategies found in the literature. However, measuring the operational performance in one scenario cannot possibly be considered equivalent to measuring swarm intelligence. Indeed, a system displaying high levels of operational performance may not necessarily have swarm intelligence. Take, for example, a multi-robot system tasked with retrieving and delivering packages to and from designated locations within a warehouse. When operating within a predictable, organized, and mostly static environment, a centralized pre-planned strategy that has been optimized for a specific warehouse is often the ideal solution (Ma et al., 2017; Bredeche and Fontbonne, 2022). Recently, attempts have also been made at developing decentralized strategies that feature reduced optimization times when the number of robots in the system is increased while maintaining similar levels of performance (Claes et al., 2017). However, both strategies would be unable to deliver the same level of performance should they be deployed in different warehouses without another round of optimization and planning. On the other hand, while swarm intelligent strategies may not perform as well as the situation-optimized solutions, they are able to adapt to changes in the environment autonomously and deliver similar levels of performances across different operating conditions. The use of an adaptivity benchmark test suite would highlight this higher level of adaptivity present within swarm intelligent MRS.

### 4.2 Testing narrow and general swarm intelligence

As previously stated, current MRS are capable of displaying narrow adaptivity, where a system is able to carry out one specific task in a dynamic environment. As such, to test for adaptivity, the system must be able to demonstrate that it is able to perform over a possibly wide range of different environmental conditions for a given class of problems. For example, in a target tracking scenario, an MRS using an adaptive strategy should be able to maintain a certain level of performance should there be changes in the number of targets to be tracked, target speed, target movement profile, etc. (Esterle, 2018a; Kwa et al., 2020; Kwa et al., 2021; Kwa et al., 2022b). In many engineered systems, there often exist performance indicators and metrics, such as those for first- and second-order linear time-invariant (LTI) controllers that allow a system’s properties to be fully understood by its users and also facilitate its comparison to other similar systems. Similar benchmark frameworks and characteristic graphs could also be developed for MRS that could reveal how a system’s performance is expected to evolve over varying environmental conditions, thereby giving an indication of a system’s level of adaptivity.

**FIGURE 2:**
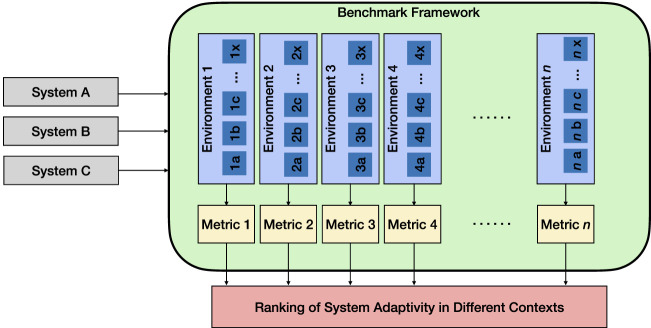
Flowchart describing our proposed benchmark framework. The systems to be compared are tested in multiple environments (environments 1, 2, …, *n*), each with different environmental parameters (*a*, *b*, …, *x*). Their performances are determined by various metrics specific to each environment, thereby allowing the ranking of the systems’ adaptivity in different contexts.

To progress from narrow adaptivity toward general swarm intelligence, the community should focus its efforts on developing systems with general adaptivity. This will endow systems with not just the ability to adapt to different environmental conditions but also with the ability to adjust their behaviors to accomplish different tasks, e.g., changing behaviors from a set suited to collective mapping to another set suited to target tracking. This taps into the central concept of transferability, where one can evaluate the system’s ability to operate in different scenarios while maintaining its level of performance. Measuring general adaptivity requires more than one class of benchmark environment. In view of this, a suite of benchmark tests should be developed and implemented, similar to the Procgen Benchmark proposed and developed by OpenAI for the testing of reinforcement learning agents (Cobbe et al., 2020). Specifically, with the benchmark, developers are able to measure how quickly their agents learn generalizable skills in 16 procedurally generated environments, in effect measuring their agent’s level of general adaptivity. It is worth adding that these 16 environments have been selected to assess different characteristics. In our proposed benchmark framework, as shown in Figure 2, each benchmark test environment would be associated with its own set of environmental parameters and performance metrics, thereby allowing the user to determine which system is more adaptive in any of the given contexts.

Although more standardized benchmarks can improve the development of adaptivity in swarms, it is not a panacea. The existence of such benchmarks may give clear targets and methods to evaluate a given set of strategies, which may inadvertently lead to other factors not included by the benchmark to be ignored. As stated by Raji et al. (2021), there is no test dataset or environment that can capture the full details and complexity of all the possible scenarios in which an MRS can be deployed. Hence, any implemented benchmark framework must, like general swarm intelligence algorithms, evolve to prevent what is essentially the overfitting of strategies to particular benchmarks. Such evolving environments have already been implemented by Cobbe et al. (2020); the benchmark uses procedurally generated environments, essentially offering a “near-infinite supply of randomized content,” preventing an agent from memorizing an optimal policy. The ultimate goal of any proposed benchmark framework should not be to declare if an algorithm has attained general adaptivity but to enable practitioners to understand how systems are able to adapt to their immediate surroundings and compare different systems while they are executing similar tasks.

## 5 Discussion

Many parallels can be established between the development of artificial general intelligence and general swarm intelligence. This includes the search for a general (swarm) intelligence algorithm and a fixation of the community on developing tailor-made strategies for very specific circumstances. However, we believe that these two are contradictory objectives. Developing environment-specific strategies will essentially maximize the performance of a swarming MRS for a particular set of conditions. However, these high levels of performance would most likely falter should the conditions deviate from those considered at the design stage. A system designed for adaptivity would avoid such declines in performance when changing conditions, and thereby, moves us closer to developing general swarm intelligence algorithms.

We believe the key to unlocking these general swarm intelligence algorithms is through increasing the system’s level of adaptivity. This would allow a swarm to categorize and understand its environment, thereby giving it the ability to change its collective behavior to match its changing goals in dynamic environments. As previously mentioned, such a system needs to be multipurpose and be able to optimize its own behavioral parameters, or even develop new ones, to achieve its new objectives in the most efficient manner (Birattari et al., 2020; Hasbach and Bennewitz, 2022). For example, in the absence of any targets, a swarm with general SI originally performing a target tracking task may find it more appropriate to transition to carry out area mapping and, therefore, optimize its actions and behavior for this new task. Should a single target appear in the environment, the system may even deem it appropriate to carry out both tracking and mapping tasks concurrently.

As a first step toward general SI, adaptivity must be embedded into the system design process, essentially allowing the system to learn to be adaptive. From the AI standpoint, this means that the robotic system is capable of dealing with an open world, such that techniques to enable adaptivity during operation—and not at the design stage—will be the key. To this end, we expect designers to train their agents in gradually more open environments using multi-agent reinforcement learning (MARL), allowing for the effects of dynamic environmental factors to be included in the training process. Indeed, several research groups have already started using MARL techniques to develop policies for their swarming agents in dynamic environments (Kouzehgar et al., 2020; Wang et al., 2022a; Wang et al., 2022b; Kouzeghar et al., 2023). Learning from demonstration, experience replay, and transfer learning offer promising opportunities to exploit prior knowledge, e.g., from another domain or task (Karimpanal and Bouffanais, 2018; Karimpanal and Bouffanais, 2019). However, these powerful techniques will have to be extended to multi-agent systems.

Although current MARL-trained swarms only focus on achieving a single objective, enabling a swarm to switch between tasks or carry out tasks simultaneously can possibly be achieved by setting the swarm’s priorities. Doing so would allow a system to identify environmental cues to trigger the switch between scheduled tasks, thereby allowing it to ascertain when and how to accomplish different tasks as it learns and adapts to its environment. However, to attain such a high level of adaptivity, a system needs to account for a wide range of factors and missions while simultaneously being able to avoid reacting to false alarms. It is not feasible for MARL practitioners to handcraft a reward function and perform hyperparameter tuning for a system with so many parameters and goals over all possible environments. As such, further advances to swarm adaptivity may come from the newly established field of automated reinforcement learning (AutoRL), essentially enabling a swarming MRS to train itself, i.e., self-learn (Faust et al., 2019; Parker-Holder et al., 2022).

Given the need to design for adaptivity, it would also be beneficial to quantify this level of adaptivity and see how different swarming systems compare with each other. Similar to the testing of “standard” artificial intelligence and computational optimization algorithms, there is a critical need for the swarm intelligence community to implement a suite of evolving benchmark problems, along with metrics to evaluate the performance of swarming systems, with a focus on adaptivity. Although there are works that compare the performance of different swarming algorithms, such comparisons are usually incomplete, with the two strategies usually being tested within a narrow range of environmental conditions and tasks. Since it has previously been shown that MRS performances are highly sensitive to the demands of the task and those of the operating environment, the current limited form of testing may give a false impression that one swarm strategy is able to outperform another over all conditions and settings. Therefore, an implementation of a standardized benchmark framework, consisting of a suite of benchmark problems, would allow for a more complete and accurate comparison of different swarm algorithms and also contribute to faster algorithm development and evaluation.

## Data Availability

The original contributions presented in the study are included in the article/Supplementary Material; further inquiries can be directed to the corresponding author.
